# Service, training, mentorship: first report of an innovative education-support program to revitalize primary care social service in Chiapas, Mexico

**DOI:** 10.3402/gha.v7.25139

**Published:** 2014-11-03

**Authors:** Andrew Van Wieren, Lindsay Palazuelos, Patrick F. Elliott, Jafet Arrieta, Hugo Flores, Daniel Palazuelos

**Affiliations:** 1 Division of Global Health Equity, Department of Medicine, Brigham and Women's Hospital, Boston, MA, USA; 2 Partners in Health/Compañeros en Salud, Boston, MA, USA; 3 Department of Global Health and Social Medicine, Harvard Medical School, Boston, MA, USA

**Keywords:** global health, primary care, social service, medical education, health systems strengthening, underserved, Mexico, Latin America

## Abstract

**Background:**

The Mexican mandatory year of social service following medical school, or pasantía, is designed to provide a safety net for the underserved. However, social service physicians (pasantes) are typically unpracticed, unsupervised, and unsupported. Significant demotivation, absenteeism, and underperformance typically plague the social service year.

**Objective:**

Compañeros en Salud (CES) aimed to create an education-support package to turn the pasantía into a transformative learning experience.

**Design:**

CES recruited pasantes to complete their pasantía in CES-supported Ministry of Health clinics in rural Chiapas. The program aims to: 1) train pasantes to more effectively deliver primary care, 2) expose pasantes to central concepts of global health and social medicine, and 3) foster career development of pasantes. Program components include supportive supervision, on-site mentorship, clinical information resources, monthly interactive seminars, and improved clinic function. We report quantitative and qualitative pasante survey data collected from February 2012 to August 2013 to discuss strengths and weaknesses of this program and its implications for the pasante workforce in Mexico.

**Results:**

Pasantes reported that their medical knowledge, and clinical and leadership skills all improved during the CES education-support program. Most pasantes felt the program had an overall positive effect on their career goals and plans, although their self-report of preparedness for the Mexican residency entrance exam (ENARM) decreased during the social service year. One hundred percent reported they were satisfied with the CES-supported pasantía experience and wished to help the poor and underserved in their careers.

**Conclusions:**

Education-support programs similar to the CES program may encourage graduating medical students to complete their social service in underserved areas, improve the quality of care provided by pasantes, and address many of the known shortcomings of the pasantía. Additional efforts should focus on developing a strategy to expand this education-support model so that more pasantes throughout Mexico can experience a transformative, career-building, social service year.

Ever since the President of Mexico and the Dean of the Universidad Nacional Autónoma de México established an agreement in 1937, graduating Mexican medical students have been required to complete a year of primary care social service (called pasantía) before obtaining their full medical license (1, 2). Distributing social service physicians (pasantes) throughout Mexico is intended to provide a safety net for the underserved. In reality, however, pasantes are typically unsupervised and unpracticed (meaning that they are both inexperienced and have not undergone enough mentored training to adequately practice without further oversight). In addition, they are often distracted by pending residency entrance exams, and regularly try to secure placements in more comfortable urban environments (3, 4). When they are assigned rural sites, lack of support, absenteeism, and underperformance often define their experience 4–6).

Compañeros en Salud (CES), the Mexican branch of Partners in Health (PIH), is a non-governmental organization (NGO) whose mission is to build a model of excellence in rural primary care in partnership with the Ministry of Health (MOH). The founders of CES have each worked in the Sierra Madre Mountains of the state of Chiapas – one of the poorest areas in Mexico – for 5–10 years, and found that access to primary care was limited by physician shortages (5). Although Mexico's health system reform in 2003–2004 expanded public health insurance coverage to the poorest Mexicans, its full promise has yet to be realized, as well-trained and logistically supported health care professionals are often not available to provide high-quality care in the rural areas where many of the poorest Mexicans live 7–9) . In an effort to expand effective access to care in the Sierra Madre region, CES began to recruit pasantes to work in MOH clinics that previously lacked a physician.

CES recognized that working with social service physicians also provided an opportunity to address known shortcomings of the pasantía through creating a transformative educational experience. The CES approach to transformative education was inspired by a call to action from the Lancet Commission for Health Professions Education asking educators to not just inform students by imparting knowledge and skills or form them through teaching professional values, but rather transform students through developing leadership attributes and enlightening them as agents of change (10). In this transformative spirit, CES staff and US physician collaborators created an innovative education-support program that aims to: 1) train pasantes to effectively deliver primary care, 2) expose pasantes to central concepts of global health and social medicine, and 3) foster continuing medical education and career development of pasantes. This paper describes the components, evaluation, and insights of the first 2 years of the CES education-support program.

## Methods

### Pasante recruitment

CES staff (LP, JA, HF, and DP) worked with faculty and a student group at the Monterrey Institute of Technology Medical School (ITESM) – and subsequently with several other medical schools – to identify graduating medical students interested in completing their pasantía in Chiapas. Initial connections between CES and the ITESM were established through several of the CES staff being alumni or having previous affiliations with ITESM. CES staff have subsequently connected with global health-oriented student groups from medical schools throughout Mexico at national and international conferences, which has facilitated recruitment of pasantes from medical schools in many different areas of Mexico, including Nuevo León, Mexico City, Querétaro, Durango, Veracruz, Tamaulipas, Chihuahua, and Puebla. During recruitment, CES sought candidates with the following characteristics: academic excellence, interest in global health and social medicine, perceived ability to adapt to an impoverished rural community, and expressed desire to provide primary care to marginalized patients. To ensure that income is not a barrier to participation, CES offers pasantes a matching stipend to that provided by the government for a total combined stipend of about US$380 monthly (4).

### Site selection

CES staff (LP, HF, and DP) identified communities in the Sierra Madre region of Chiapas with public clinics that lacked a full-time physician. Local and state MOH authorities granted CES permission to recruit pasantes to these clinics and provide a package of training and logistical support. CES staff then met with community leaders to introduce the organization, offer a pasante to the local clinic, and request collaboration (including willingness to help pasantes find room and board). Two communities chose to participate in February 2012, and an additional four joined in August 2012. CES aims to expand to a total of 10 clinics by the end of 2015.

### Program development and components

The education-support program strives to transform the entire pasantía into a learning experience. The program harnesses both clinic and classroom time with five fundamental components: 1) monthly multi-day supportive supervision visits by CES staff, 2) biannual multi-week mentorship by medical resident and attending physician volunteers, 3) access to clinical information resources such as locally tailored evidence-based treatment algorithms and UpToDate©, 4) a monthly 2–3 day interactive seminar utilizing adult learning best practices, and 5) improved functioning of the clinics where pasantes work. The CES approach to both patient care and pasante mentorship utilizes a style called ‘accompaniment’, which emphasizes solidarity and collective problem solving.

#### Supportive supervision

CES hires a select group of graduated pasantes and external applicants as supervisors (in a ratio of approximately one supervisor per three to four pasantes) to provide holistic support for the next generation of pasantes. CES uses a model of supportive supervision informed by the World Health Organization (WHO) concept: supervision is helping to make things work, rather than checking to see what is wrong (11). Supervisors visit each site for about 3 days per month and review a portfolio of functions with the pasante, including troubleshooting difficult cases, clinic management, facilitating referrals, and negotiating community relations. The pasante and supervisor jointly identify priority areas for improvement and make plans for how to address them. The system encourages performance by recognizing and praising when standards are met, and using a supportive rather than primarily punitive approach when they are not.

#### Clinical mentorship

Throughout the year and with intensified focus when pasantes first arrive in February and August, volunteer residents from the USA and Mexico of different specialties (e.g. Family Medicine, Internal Medicine, Pediatrics, Obstetrics/Gynecology, Psychiatry, and Neurology) pair with pasantes to provide intensive bedside teaching. Clinical mentors do not directly provide care, but rather precept and reinforce core skills, talking through each patient's diagnosis, counseling, and treatment with the pasante.

#### Clinical information resources

In an effort to promote high-quality and standardized care among pasantes, CES developed a series of clinical algorithms to guide pasante decision-making for common primary care problems (e.g. acute cough, dysuria, hypertension, diabetes mellitus, etc.). CES algorithms respect official MOH norms, incorporate likelihood ratios for history and physical exam findings, and use principles from care delivery value chains to encourage pasantes to provide evidence-based and locally tailored care 12–14). CES also provides pasantes with free access to UpToDate©.

#### Interactive seminar

CES designed a monthly course utilizing adult learning best practices such as building on existing knowledge, using a ‘teach-back’ method, and project and case-based exercises. ITESM accredited the seminar as a 12-month certificate course in Global Health and Social Medicine. The curriculum has undergone two rounds of formal evaluation in an effort to improve its contents and delivery. Because new pasantes arrive to work with CES every 6 months, fundamental components of the curriculum are delivered every 6 months, allowing pasantes in the second half of their year to take on a ‘teach-back’ role, whereas other components of the curriculum are delivered once every 12 months.

The course content focuses on three main areas: 1) clinical medicine taught through problem-based learning cases, case presentations done in ‘morning report’ style, and use of ultrasound and other diagnostic tests in the primary care setting; 2) global health and social medicine delivered via case-based health systems analysis, socioeconomic determinants of health, policy, and cultural competency and humility; and 3) quality care delivery (e.g. clinical leadership skills, team-based care, quality improvement projects, morbidity and mortality review, feedback sessions, and humanistic curriculum). CES staff, volunteer residents, guest speakers, graduated pasante supervisors and, in many cases, pasantes in the latter half of their pasantía all teach different sessions of the course, with all sessions supervised by CES leadership and often using a team-teaching strategy. The 2–3 day monthly seminar also provides pasantes an opportunity to build community and support each other throughout the challenging social service year. As part of the course, pasantes also receive complementary EXARMED© study materials for the Mexican residency entrance exam (ENARM).

#### Improved function

CES pasantes leave their assigned communities for approximately 7–8 pre-planned consecutive days each month, 3–4 days of which are spent between the 2–3 day educational seminar at the CES office, 1 day of turning in paperwork at the jurisdiction offices, and 1–2 days of travel time. The remaining 3–4 days are assigned as vacation. This model of concentrating days away from the community purposely differs from the traditional model of pasantes having 1–2 days off a week, because the long trips required to exit and then re-enter isolated rural communities tend to diminish actual time off and promote absenteeism (4, 5). CES-supported clinics also receive supplemental supplies to ensure there are no stock-outs of essential medicines, diagnostic tests, and basic infection control supplies.

### Motivating and retaining pasantes

Adequately supporting pasantes during their social service year requires many complex and interdependent investments. In a systematic review of factors that help motivate and retain health workers in underserved areas, Willis-Shattuck found that seven critical strategies combat ‘brain drain’ and promote ‘brain gain’ (15). Table 1 provides examples of how CES has learned to address these seven areas programmatically. Furthermore, because new pasantes coming from different educational backgrounds have varied levels of preparedness for both the clinical and psychosocial demands of the social service year – similar to new interns starting residency programs – CES strives to quickly identify pasantes who require additional support to catch up to their peers and then provides supplemental supportive supervision and clinical mentorship during the first month of the pasantía.

**Table 1:** Seven key motivating factors (15) and corresponding CES strategies to attract and retain pasantes

Motivating factor	CES strategies
Financial rewards	-Match pasante stipend, increasing the total from approximately ~US$190 to ~US$380 monthly
Career development	-Provide opportunities for pasantes to work in management roles within CES after completing the pasantía -Provide opportunities for pasantes to visit other Partners in Health sites
Continuing education	-Monthly CES course -Supervisor and resident accompaniment in clinic -Clinical information resources such as clinical algorithms and access to UpToDate©
Work environment	-Providing electronic medical record and computers for clinic notes and for automatic generation of government paperwork that previously took hours to complete by hand
Resource availability	-Working with Mexican government to prevent ‘stock-outs’ of essential medications -Supplementing medication stocks -Helping pasantes refer complex patients
Workplace management	-Teaching pasantes how to work as part of and lead a clinical team -Regular visits by supervisors and leadership to help build teamwork among clinic staff -Advising pasantes on how to manage patient flow and limit wait times
Recognition/appreciation	-Providing a certificate through the ITESM for the Global Health and Social Medicine course -Supporting pasantes to integrate with the communities they serve

### Survey instrument

The authors collected data about the education-support program and its influence on pasantes using pre-, mid-, and post-intervention surveys that ranged in length from 18 to 23 questions. The surveys incorporated both Likert scale and open-ended questions, and explored the following areas: pasantes’ self-reported medical knowledge (overall medical knowledge, and specialized knowledge in Internal Medicine, Pediatrics, Obstetrics/Gynecology, and Emergency Medicine); clinical leadership skills and understanding of the Mexican health care system; preparedness for the ENARM; residency and career plans (planning on applying for residency, type of medicine planning to pursue, desire to work with the poor or underserved); best and worst experiences as a pasante; best aspects and recommended changes for the education-support program; and degree of satisfaction with the CES program. Content of the three surveys was similar to facilitate measuring changes throughout the year. The survey instruments were written by AVW, LP, and DP in English and then translated into Spanish by a bilingual native Spanish-speaking CES volunteer.

### Study design, data collection, and analysis

The present study was conceived as a case study of pasantes completing their social service requirement as part of the CES program. The data reported were collected from February 2012 through August 2013. The data reflect completed pre-, mid-, and post-intervention questionnaires for six pasantes, representing the first two classes of pasantes to complete the CES education-support program. Data are not reported for two pasantes who did not complete the study, one who left the CES-affiliated pasantía due to a desire to be closer to family, and one who had incomplete data. To put the current size of the CES program in context, 263 of 932 (28.2%) public primary care clinics in Chiapas are staffed exclusively by pasantes (4). Surveys were distributed to pasantes during a designated time at the seminars and pasantes were given as much time as needed to complete them. Pasantes wrote a code that only they would understand on their three surveys in order to concurrently track data and preserve anonymity. AVW, LP, and DP performed data analysis, which included conducting descriptive statistics on quantitative data, and identifying predominant themes and supporting quotations from qualitative data. This study was reviewed by the Partners Healthcare Institutional Review Board (Brigham and Women′s Hospital) in Boston, MA, and was given IRB exemption.

### Evaluation of the program

CES staff evaluates the CES education-support program on both an informal continuous basis and also at designated formal intervals at the end of each pasante cycle (every 6 months in January and July). Pasantes evaluate the program through the aforementioned pre-, mid-, and post-intervention questionnaires and through informal feedback obtained by staff and volunteers throughout the social service year. CES staff members verbally evaluate the program during regularly scheduled monthly meetings with CES leadership. CES volunteers provide either verbal or written evaluations of the program at the conclusion of their volunteer period. CES then uses feedback from pasantes, staff members, and volunteers to implement new changes to both the educational and support aspects of the curriculum at the beginning of each new pasante cycle (every 6 months in February and August). CES implements changes to the education-support program using a Plan-Do-Study-Act model, in which feedback is reviewed by CES leadership to form plans for change, which are then implemented, subsequently studied in the next semester's review process, and then the change plans are either adopted, adapted, or abandoned.

## Results

The six pasantes who completed the study ranged in age from 22 to 26 years, included three men and three women, had all completed required coursework at an accredited medical school in Mexico making them eligible to complete their social service requirement, and all had completed the pasantía as part of the CES education-support program in Chiapas.

### Knowledge and skills

**Fig. 1:**
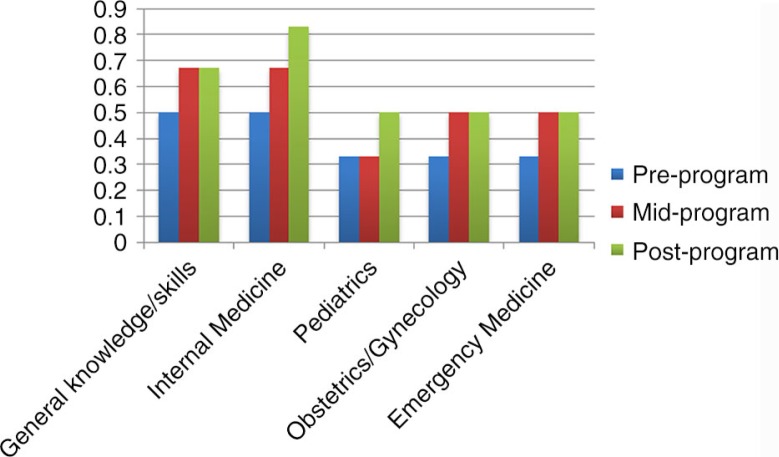
Percent of pasantes reporting good or very good general clinical knowledge and preparedness to practice several medical specialties before, halfway through, and at the end of the CES education-support program.

Overall, pasantes reported that their medical knowledge and clinical skills improved while completing the education-support program during their social service year in Chiapas. Figure 1 demonstrates how pasante self-report of general clinical knowledge and preparedness to practice Internal Medicine, Pediatrics, Obstetrics/Gynecology, and Emergency Medicine changed throughout the course of the education-support program. When asked to reflect on changes in knowledge and skills during the pasantía, one pasante remarked, ‘I have gained greater medical knowledge because of the accompaniment I received from residents, the feedback I received from patients, and from being able to longitudinally observe the patients I treated in my community’. Another observed, ‘My abilities to create differential diagnoses and treat patients are constantly improving because of the feedback and accompaniment I have received from the CES staff, residents, and volunteers’.

Pasantes reported that clinical leadership skills and understanding of the Mexican health care system also improved throughout the course of the education-support program. The percentage of pasantes who reported feeling well or very well prepared to lead a primary care clinic increased from 50% at the beginning of the program to 83% by the end of the program. Whereas 67% of pasantes reported understanding the organization or hierarchy of the Mexican health care system well or very well at the beginning of the program, 100% of pasantes felt that way by the end of the program.

Throughout the course of the education-support program, pasante report of perceived preparedness for the Mexican residency entrance exam (ENARM) decreased and, by the end of the year, most pasantes had decided not to take the ENARM in the year following their pasantía. Although 100% of pasantes believed the pasantía would have a positive or very positive effect on their performance on ENARM at the beginning of the program, by the end of the year 83% of pasantes had decided against taking the ENARM in the year following their pasantía.

Notably, pasantes reported learning more than just rote clinical knowledge. Pasantes also recalled developing more nuanced clinical skills – such as the ability to use a referral network for more complex cases – and beginning to appreciate and address the social determinants of health. One pasante reflected, ‘I found that I was actually able to accurately diagnose and treat the majority of my patients; for the more complicated cases in which I did not know what to do, I fortunately had the support of the entire CES team to first figure out the diagnosis and then implement the best treatment plan’. Another explained, ‘I am beginning to understand how to break the cycle of poverty and disease by treating diseases that were previously incurable in the Sierra, diseases that affected whole generations and society as a whole’.

### Career goals and plans

Pasante career goals and plans changed in several ways throughout the course of the education-support program. When asked if they were considering a career in primary care at the beginning of the year, 33% responded ‘probably no’, 33% ‘undecided’, and 33% ‘probably yes’. By the end of program, 67% of pasantes reported they were either definitely or probably not considering a career in primary care, but 33% reported they definitely were considering a career in primary care and no pasantes remained undecided. The percentage of pasantes who reported a desire to work primarily with poor and underserved populations during their career increased from 83 to 100% between the beginning and end of the program. By the end of the year, 83% of pasantes felt the social service year had a positive or very positive effect on their career goals and plans. One pasante detailed this transition as follows: ‘The trajectory of my life has taken a significant turn after working with CES. I would like to work with different communities, especially the poor communities where CES works’. Another pasante reflected, ‘Before I arrived to work with CES, my life plan was set. Now I'm not sure what I want to do. The only thing I'm sure of is that, whatever happens, I want to continue collaborating with CES in the short- and long-term’.

### Satisfaction with pasantía

When asked at the end of the year, 100% of pasantes reported they were satisfied with their experience as a pasante and that they were glad they had done their social service year in CES-supported government clinics in Chiapas. When asked why they were satisfied with the pasantía, one pasante wrote, ‘I grew personally and professionally’, whereas another pasante explained ‘[the year] provided me a totally new perspective regarding professional opportunities that I had never imagined’. At the end of the year, 67% of pasantes reported that they had achieved their goals for the pasantía. Among the pasantes who reported achieving their goals, cited reasons why included: ‘I learned how to practice medicine and feel confident as a doctor’, and ‘I completed all of my goals and even others I formed during the year’. One of the pasantes who denied having achieved his or her goals for the year explained: ‘I gained many clinical skills and helped many patients. However, I did not study for ENARM as much as I had hoped’.

### Satisfaction with education-support program

Table 2 shows highlights of what pasantes felt were the best and worst aspects of both the overall pasantía and the CES education-support program. Regarding the overall pasantía, pasantes appeared to appreciate the following: gaining clinical knowledge and confidence, developing relationships with their patients over time, seeing their patients improve, having resident and supervisor accompaniment, attending the monthly global health seminar, working on quality improvement projects, and exploring a new area of Mexico. In critiquing the overall pasantía, pasantes cited the following: running out of available treatment options, patients not following up as requested, social isolation in the communities, frustration and fatigue throughout the course of the year, limited collaboration with nurses in the clinics, and growing pains of CES as a new organization. When asked to describe the best aspects of the CES education-support program, pasantes praised: the program's global health and social medicine focus, its case-based approach, the program being new and unique in Mexico, CES always striving to improve the program, resident and supervisor accompaniment, the ITESM certificate awarded at the conclusion of the program, and the program being tailored to the local reality of Chiapas. Asked to identify the worst aspects of the CES education-support program, pasantes criticized: the seminar's classes being too long and dense, the curriculum being poorly defined, the teaching approach being too informal, too many different instructors teaching the courses, too much focus on quality improvement, and limited distribution of written resources.

**Table 2:** Pasante report of best and worst aspects of the overall pasantía, and best and worst aspects of the CES education-support program

Best aspects of overall pasantía:	Worst aspects of overall pasantía:
Gaining clinical knowledge and confidence Seeing patients improve Long-term relationships with patients Resident and supervisor accompaniment Traveling to new area of Mexico Monthly global health seminar Working on quality improvement projects	Running out of available treatment options When patients did not follow up as requested Being alone in the community Frustration and fatigue Growing pains of CES as a new organization Limited collaboration with nurses
Best aspects of education-support program: Global health and social medicine focus Case-based approach Program is new and unique in Mexico CES is always working to improve program Resident and supervisor accompaniment Certificate awarded at end of course Locally oriented to Chiapas	Worst aspects of education-support program: Classes are too long Classes are too dense Curriculum is not defined well enough Too informal Too many different people teaching classes Too much focus on quality improvement Limited distribution of written resources
	

## Discussion

Building on changes introduced by the Mexican health system reform of 2003–2004, the Mexican health care system has recently been celebrated for providing universal health coverage to all Mexicans (7, 8). Despite this high level of enrollment, gaps in effective coverage persist (16). Truly achieving equitable access to high-quality care within Mexico will require an ongoing commitment to ensuring primary care providers arrive at the most underserved regions of Mexico and are provided logistical and training support so that they can perform at a high level (17).

In Mexico, despite the fact that pasantes are still technically considered students until they complete their social service year, as many as one-third of public primary care clinics are staffed exclusively by pasantes and nearly three-quarters of pasantes are assigned to rural health centers (4). This represents a large work force addressing rural health needs, but there is a growing concern that, similar to interns beginning residency, Mexican pasantes would provide higher quality and safer care if they were supervised, supported, and continued to receive education during the social service year (4, 5). Innovative approaches to supporting and training pasantes during their social service year are sorely needed to help turn Mexico's universal enrollment into true universal effective coverage.

Given the limited number of studies evaluating strategies to address human resources concerns and strengthen primary health care systems in low- and middle-income countries (18, 19), this program and its evaluation offer important insights to those interested in strengthening the primary health care system in Mexico and wherever social service providers are dispatched. Our results suggest that the CES education-support program has several important strengths and insights.

The results of this study show that the CES education-support program is a transformative experience for participants. The Lancet Commission for Health Professions Education has called for efforts at medical education reform to be guided by two main principles: transformative (rather than only informative or formative) learning, and interdependent and interdisciplinary education (10). Involvement in the CES program fundamentally changed the way the pasantes considered their future; all experienced an increased commitment to work with the poor and underserved in the future. In addition, they reported greater clinical leadership skills and a more nuanced understanding of the Mexican health care system. The Lancet Commission for Health Professions Education has highlighted several programs that model transformative education, such as the Public Health Foundation of India which uses a similar public-private partnership as the CES model to transform public health professionals during a 12-month program that couples field work with classroom learning (10). CES aims to draw from both internal program evaluation and lessons learned from others in an effort to constantly improve the CES program and make it as transformative as possible for pasantes. Although the MOH did provide pasantes with some basic supervision and training before development of the CES program, the public-private partnership between the MOH and CES has resulted in the development of a program that now teaches pasantes leadership skills and how to potentially be agents of change within the Mexican health care system.

The findings also highlight the importance of classroom training that goes beyond technical proficiencies to integrate systems-level concepts of global health, social medicine, and quality improvement. The Global Health and Social Medicine course appears to instill a sense of mission within pasantes, thus invigorating the care they provide to underserved patients. Interest in global health is rising to unprecedented levels among medical trainees, and seems to represent a revitalization of the sense of mission and altruism that is core to the medical profession (20, 21). Inviting residents and attending physicians from Mexico, the USA, and elsewhere to offer clinical ‘accompaniment’ of pasantes represents a powerful way in which interest in global health can be harnessed to improve training for not only Mexican providers but also the visiting physicians themselves (10, 19). Efforts to create effective and sustainable approaches to pasante training and mentoring will benefit from new partnerships that have not been previously considered, including partnerships between the public sector, private NGOs, and academic institutions.

Although critically evaluating this type of education-support program will inform future efforts to support and educate pasantes, we must also consider how public policy changes could influence pasante behaviors and promote effective universal access (15, 19). Indeed, some limitations of the pasantía model may be better addressed through public policy changes than educational programs alone. For example, entrance to residency programs in Mexico is determined largely by the ENARM test, typically taken after the social service year. Despite providing complementary study preparation materials for ENARM, pasantes’ self-reported preparedness for the ENARM exam decreased during the social service year and most pasantes had decided to either postpone or not take ENARM by the end of the pasantía. We suspect this phenomenon is the result of clinical responsibilities precluding pasantes from having sufficient study time. Perhaps a more realistic and transformative approach is that used within the Chilean social service system. In Chile, rather than having to worry about how social service time will negatively affect performance on residency entrance exams, pasantes who complete their social service in rural undeserved areas are awarded additional points on the medical residency entrance exam (4).

Another example of how public policy changes could improve the social service year is by securing support within the most recent Mexican health reform to expand education-support programs for pasantes within the public sector. Although the CES program has created a unique educational environment within six government-run clinics, the state of Chiapas alone has 263 primary care clinics that are staffed exclusively by pasantes (4). The CES program relies upon private donations and is run by a NGO, and is thus likely not sustainable at a large scale. We believe that the question is not whether there is enough money to better train and support the providers who care for the most underserved Mexicans, but rather whether sufficient political will exists to align funds for this purpose. As the Lancet Commission for Health Professions Education points out, despite the labor-intensive nature of medicine, only 2% of global health expenditures are spent on training – a percentage that is even smaller in low-income countries (10). Furthermore, in his critical appraisal of the pasantía in Mexico, Nigenda proposes that, with relatively modest financial investment, the Mexican government could feasibly hire licensed and fully trained physicians to work alongside pasantes, guiding and training them during their social service year (4). In fact, some Mexican districts already provide pasantes with limited intermittent training and supervision. However, our data support the deployment of more comprehensive training and support packages that convert the social service year into a transformative experience.

### Study limitations

Our study results have several important limitations. First, because we did not incorporate a comparison group of pasantes not participating in the education-support program into the study, the results could simply show the effect of completing a social service year rather than that of the education-support program. Furthermore, because many of the survey questions were retrospective about experiences during the preceding months, the results are subject to recall bias. Finally, because our sample size of pasantes is small (*N*=6), our quantitative results should be interpreted as suggesting possible trends rather than establishing statistically significant patterns.

### Opportunities for future work

Ultimately, if we are to engage in true health systems strengthening, we will need to develop a strategy to fully transition the CES education-support program to the public sector (22). Large-scale expansion of this type of education-support program with public funding, however, should only be undertaken after more rigorous studies have demonstrated a clear benefit of the education-support program by directly comparing it to the social service year alone. Notably, recruitment of pasantes for the program and study was likely facilitated by affiliations with ITESM, PIH, and Harvard Medical School – all well-known and respected private institutions – and, thus, efforts to expand this type of program within the public sector would need to consider how those organizational affiliations could either be maintained at larger scale or explore other types of recruitment incentives. However, there is an expanding body of literature indicating that a large percentage of medical students throughout the world are interested in global health suggesting that, if the pasantía in Mexico (and elsewhere in Latin America) could be appropriately re-branded as a global health effort, enthusiasm for the social service requirement might surge at the national and international level (20). Finally, subsequent studies of pasante education and support should attempt to move from pasante perceptions of outcomes, such as clinical knowledge and skills, to more robust measures such as test scores, and peer or patient evaluation of pasantes.

## Conclusions

Guaranteeing universal access to high-quality health care for the poor in Mexico requires not only financial and organizational reform, but also achieving an equitable distribution of well-trained and operationally supported health care providers. In Mexico and much of Latin America, the poor depend largely upon health care services provided by pasantes who are often inadequately prepared to practice independently. Efforts that focus on training and mentoring during the social service year represent a unique opportunity to improve the care that the poor and vulnerable receive, and build a cadre of providers motivated to serve them. Based on our data, education-support programs that combine on-site accompaniment of pasantes, access to clinical information resources, and regular seminars appear to be an effective way to recruit pasantes to underserved areas, increase their clinical knowledge and leadership skills, and address many of the known shortcomings of the social-service year. Additional efforts could focus on fully transitioning this type of education-support program to the public sector and developing an expansion model to reach pasantes throughout Mexico and Latin America.

## Disclosures

AVW received funding for his travel, living expenses while in Chiapas, and limited supplies through a Martin Solomon Primary Care Scholarship through Brigham and Women′s Hospital Department of Internal Medicine. CES is funded primarily by donations received through Partners in Health.
